# Case Report: Phenotypic Switch in High-Grade B-Cell Lymphoma With *MYC* and *BCL6* Rearrangements: A Potential Mechanism of Therapeutic Resistance in Lymphoma?

**DOI:** 10.3389/fonc.2021.795330

**Published:** 2021-12-23

**Authors:** Hui Liu, Qi Shen, Chung-Che Chang, Shimin Hu

**Affiliations:** ^1^ Department of Pathology, The Affiliated Hospital of Xuzhou Medical University, Xuzhou, China; ^2^ Department of Pathology & Laboratory Medicine, AdventHealth Cancer Institute, Orlando, FL, United States; ^3^ Department of Hematopathology, The University of Texas MD Anderson Cancer Center, Houston, TX, United States

**Keywords:** High-grade B-cell lymphoma, MYC, BCL2, BCL6, plasmablastic lymphoma, lineage switch, plasticity, therapeutic resistance

## Abstract

Lineage switch between myeloid and lymphoid in acute leukemia is well established as a mechanism for leukemic cells to escape chemotherapy. Cross-lineage transformation is also recognized in some solid tumors on targeted therapy, such as adenocarcinomas of the lung and prostate that transforms to neuroendocrine carcinoma on targeted therapy. Now lineage plasticity is increasingly recognized in mature lymphomas, such as small B-cell lymphomas transforming to histiocytic/dendritic cell sarcoma. However, there is no report of aggressive mature B-cell lymphoma switching from one histologic category to another upon targeted therapy. We report here a case of high-grade B-cell lymphoma with *MYC* and *BCL6* rearrangements relapsing as a high-grade plasmablastic neoplasm with *MYC* and *BCL6* rearrangements after R-CHOP and R-EPOCH therapy. Being aware of this rare scenario will help improve our understanding of the underlying mechanisms of therapeutic resistance and progression of lymphoma.

## Introduction

Significant advances have been made in cancer targeted therapy in the past decade. Unfortunately, some patients who undergo targeted therapy experience relapse after a brief remission. Accumulating evidence indicates that cancer cells change their identity by antigen remodeling and phenotypic or lineage switching to cope with the adverse microenvironment and achieve therapeutic resistance and tumor relapse and progression. Newly published data has shown that up to 5% of EGFR-mutated adenocarcinomas of the lung on targeted therapy transform their histologic subtype to neuroendocrine carcinoma. Similar trans-differentiation has also been reported in at least 20% of prostatic adenocarcinomas on antiandrogen therapy and some melanomas on MAPK inhibitor treatment (1).

In hematopoietic neoplasms, lineage switch is not uncommon, mostly in immature tumors, as the tumor cells have not yet fully differentiated and are multipotent like their normal counterparts. Cases of acute leukemia demonstrating lineage switch between myeloid and lymphoid lineages during therapy have been increasingly reported, particularly in those with *KMT2A*/*MLL* rearrangement and/or after CD19-targeted immunotherapies including blinatumomab and CAR-T cells (2–4). In contrast, lymphomas deriving from mature lymphoid cells are considered lineage stable. Nevertheless, evidence has emerged that mature lymphomas could transform from one lineage to another at recurrence. Patients with several types of small B-cell lymphomas, including follicular lymphoma, chronic lymphocytic leukemia/small lymphocytic lymphoma, mantle cell lymphoma, and marginal zone lymphoma, can develop clonally-related histiocytic/dendritic cell sarcomas (5–17). Recently, Zhang et al. and Kawashima et al. each reported one case of mantle cell lymphoma switching lineage to non-hematopoietic sarcoma with neuromuscular immuno-phenotypic features (18, 19). Besides the lineage switch to non-hematopoietic neoplasms in mature B-cell lymphomas, clonally-related classic Hodgkin lymphoma can be seen at relapse in patients with non-Hodgkin lymphomas including primary mediastinal large B-cell lymphoma and follicular lymphoma (FL), and vice versa (20, 21).

Among non-Hodgkin lymphomas, other than the histologic progression from small B-cell lymphoma to large B-cell lymphoma (LBCL) with a similar immunophenotype or verse visa, such as FL progressing to LBCL or LBCL relapsing as FL (22), however, there is no report so far of aggressive mature B-cell neoplasms switching from one histologic category to another. Here we report a patient with a high-grade B-cell lymphoma (HGBL) with *MYC* and *BCL6* rearrangements. The patient received multiple intensive treatments combined with Rituximab and achieved complete remission, but quickly developed a high-grade plasmablastic neoplasm with retained *MYC* and *BCL6* rearrangements. To the best of our knowledge, this is the first report of histologic subtype switch of high-grade non-Hodgkin lymphoma, which provides further evidence that fully differentiated mature B-cell lymphoma cells retain the lineage plasticity to escape chemotherapy.

## Case Description

A 42-year-old man who had a 4-year history of HIV infection and was not compliant with HAART therapy presented with night sweat and progressive lymphadenopathy in the bilateral neck and groin regions in May 2017. His complete blood count was WBC 4.17 x 10^3/^ul, Hgb 9.3 g/dL, and PLT 180 x 10^3/^ul, with a CD4 count of 161/ul. Position-emission tomography/computed tomography (PET/CT) scan showed extensive hypermetabolic lymphadenopathy involving bilateral cervical, bilateral axillary, subpectoral, mediastinal, bilateral hilar, gastro-hepatic, portacaval, periportal, retroperitoneal, mesenteric, bilateral iliac, and bilateral inguinal chains as well as diffuse hypermetabolism in the adenoidal soft tissue and palatine tonsils, hypermetabolism in the left parotid region, focal hypermetabolism in the left T8 paravertebral region, and splenomegaly.

**Figure 1:**
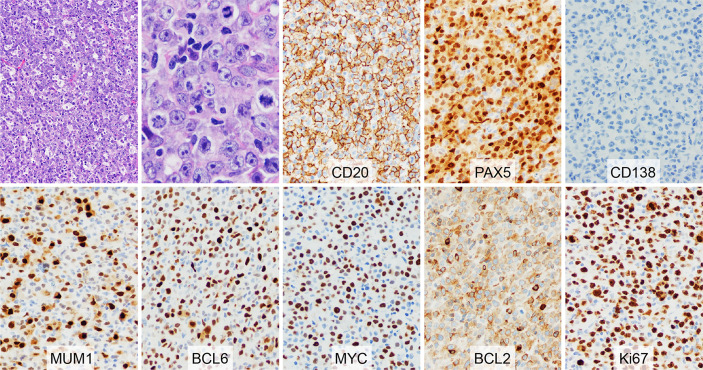
Morphology and immunophenotype of high-grade B-cell lymphoma with *MYC* and *BCL6* rearrangements at initial diagnosis. The H&E showed a large cell lymphoma with starry sky appearance. The lymphoma cells demonstrated distinct nucleoli and pale cytoplasm. Frequent mitoses were present. The lymphoma cells were positive for CD20, PAX5, MUM1 (subset), BCL6, MYC, and BCL2, and were negative for CD138. The Ki-67 proliferation rate was ~70%.

A right inguinal lymph node biopsy showed a large cell lymphoma with starry sky appearance and frequent mitoses (**Figure 1**). The lymphoma cells demonstrated frequent distinct nucleoli and pale cytoplasm. They were positive for PAX5, CD20, BCL6, MUM1 (subset), MYC, and BCL2, and were negative for CD5, CD10, CD30, CD138, cyclin D1, and EBER. The Ki-67 proliferation rate was ~70%. Fluorescence *in situ* hybridization (FISH) revealed *MYC* and *BCL6* rearrangements. No *BCL2* rearrangement was detected. PCR clonality assay showed polyclonal *IgH* rearrangement. However, the quality of DNA was low. A bone marrow biopsy was negative for lymphoma. The patient was diagnosed with HGBL with *MYC* and *BCL6* rearrangements. He was treated with 2 cycles of R-CHOP (rituximab, cyclophosphamide, hydroxydaunorubicin, vincristine, and prednisone), 4 cycles of R-EPOCH (rituximab, etoposide, prednisone, vincristine, cyclophosphamide, and hydroxydaunorubicin), and methotrexate for central nerve system prophylaxis. PET/CT scan performed in January 2018 revealed no residual disease.

**Figure 2:**
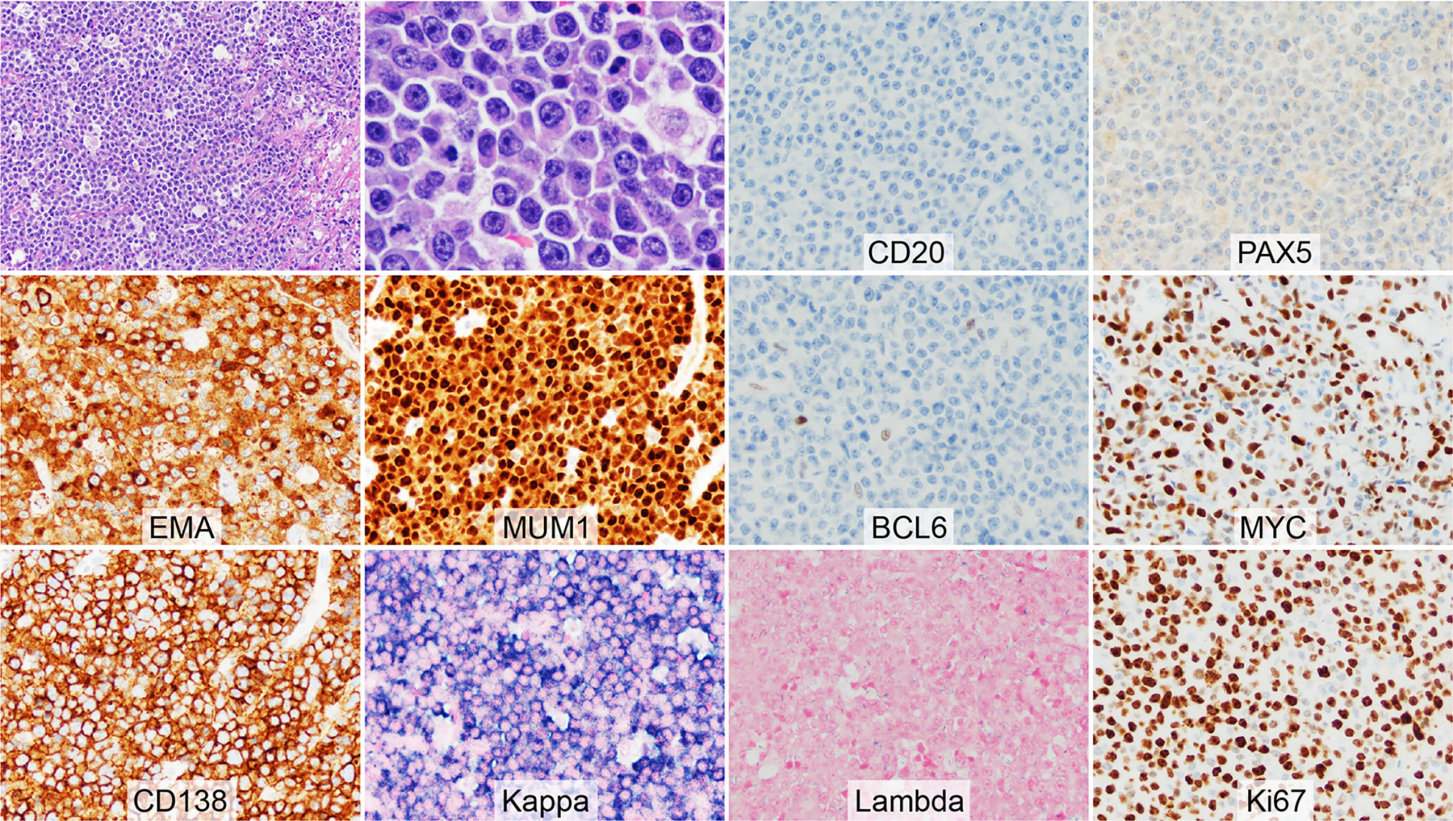
Morphology and immunophenotype of high-grade plasmablastic neoplasm with *MYC* and *BCL6* rearrangements at relapse. The H&E showed a large cell neoplasm with starry sky appearance and focal necrosis. The tumor cells demonstrated eccentric nuclei and pink cytoplasm. Frequent mitoses were present. The tumor cells were positive for EMA, MUM1 (diffuse strong), MYC, CD138, and Kappa, and were negative for CD20, PAX5, BCL6, and Lambda. The Ki-67 proliferation rate was ~90%.

Unfortunately, he developed multiple masses on the right thigh in July 2018. MRI of the right thigh without contrast showed a 23 x 10 x 9 cm mass centering in the vastus intermedius muscle and a 19 x 6 x 5 cm mass centering in the distal aspect of the sartorius muscle as well as signal abnormalities involving the greater trochanter, tibial plateau, and femoral condyle. A core biopsy of one of the thigh masses showed a large cell neoplasm with starry sky appearance and multi-focal necrosis (**Figure 2**). The tumor cells demonstrated eccentric nuclei and pink cytoplasm. They were positive for MYC, BCL2, MUM1, EMA, CD138, and Kappa, and were negative for PAX5, CD20, BCL6, CD5, CD30, CD56, ALK1, Lambda, and EBER. The Ki-67 proliferation rate was ~90%. A diagnosis of high-grade neoplasm with plasmablastic differentiation was entertained, with a differential diagnosis of plasmablastic lymphoma vs high-grade plasma cell neoplasm. Again, PCR clonality assay showed polyclonal *IgH* rearrangement based on a low-quality DNA. He was treated with 2 cycles of ICE (ifosfamide, carboplatin, and etoposide).

About 1-2 months later, PET/CT restaging revealed multiple hypermetabolic lesions within the chest, abdomen, and pelvis. An excisional biopsy of the abdominal wall mass showed a high-grade neoplasm morphologically and immunophenotypically similar to the thigh neoplasm. FISH analysis showed *MYC* and *BCL6* gene rearrangements. A diagnosis of plasmablastic lymphoma was reached. He was treated with CEOP (cyclophosphamide, etoposide, vincristine, and prednisone), then Gemcitabine plus oxaliplatin, and palliative radiation due to hydronephrosis caused by tumor compression.

Bone marrow biopsy performed in April 2019 was morphologically negative for lymphoma. However, chromosomal analysis detected in 2 of 20 cells a complex karyotype including two concurrent rearrangements involving the *BCL6* and *MYC* genes in the form of t(3;14) and t(8;14): 47,X,dic(Y;1)(q12;p12),+1,t(3;14)(q27;q32),dup(6)(p21.1p25),+7,t(8;14)(q24.2;q32),dup(12)(q13q22),add(18)(q21.3)[2]/46,XY[18]. He died in July 2019, 26 months after the initial diagnosis of HGBL.

## Discussion

We report here a case of HGBL with *MYC* and *BCL6* rearrangements switching to high-grade plasmablastic neoplasm/lymphoma with retained *MYC* and *BCL6* rearrangements at relapse. The initial lymphoma demonstrated the typical morphology and phenotype of a mature LBCL. Despite the extensive involvement of lymphoma initially, the patient responded well to chemotherapy combined with Rituximab and achieved complete remission. After a brief remission, the patient had an extensive relapse. The recurrent tumor demonstrated plasmablastic morphology and immunophenotype, losing B-cell markers (CD20, PAX5, and BCL6) and gaining plasmacytic markers (CD138, EMA, and MUM1). The presence of both *MYC* and *BCL6* rearrangements in both tumors support the clonal relatedness. Interestingly, despite retaining *BCL6* rearrangement, BCL6, a molecule that is required to be repressed for plasmacytic differentiation, was not expressed in the recurrent tumor.

From the view of development, pluripotent stem cells progressively lose their plasticity as they mature into differentiated cells and maintain lineage stability after maturation. Correspondingly, immature tumors have multi-directional differentiation potential whereas mature tumors typically show specific differentiation directions. However, this traditional view has been challenged by the growing evidence that terminally differentiated tumor cells do retain plasticity (23). These cells have the potential to modify their molecular, phenotypic, and histologic characteristics to cope with the selective stresses from the microenvironment, especially after targeted therapy. This plasticity is increasingly recognized as one of the critical mechanisms involving tumor relapse, metastasis, and therapeutic resistance (1).

In general, tumor cells change their identity along the path of differentiation, backward or forward. Differentiated tumor cells de-differentiating to an earlier stage, or even progenitor phenotype, is commonly seen in soft tissue sarcoma (23–25). Sometimes small B-cell lymphomas, such as marginal zone B-cell lymphoma, demonstrate differentiation toward terminally differentiated plasma cells, or even overwhelmed by plasma cells, particularly after therapy (26, 27). Plasmacytic differentiation requires downregulation of PAX5 and BCL6 expression mediated through upregulated MUM1 and BLIMP1 (28, 29). BCL6 is a master regulator for initiation of germinal center reaction and maintenance of germinal center immunophenotype in normal B-cells, and its downregulation is required for their exit of germinal centers. PAX5 controls the identity of B-cells and its downregulation leads to loss of expression of other B-markers, facilitating upregulation of genes for plasmacytic differentiation. Generally, B-cell lymphoma loses only CD20 expression and retains B-cell identity after Rituximab treatment. However, the recurrent lymphoma in our patient lost expression of not only CD20 but also PAX5 and BCL6. In addition, it gained expression of MUM1, CD138, and EMA, indicating a switch to plasmacytic immunophenotype. The loss of B-cell identify renders the lymphoma cells less susceptible to the chemotherapeutic regimens for B-cell lymphomas. Interestingly, PCR analyses both at initial diagnosis and at relapse failed to detect monoclonal *IgH* rearrangements based on low-quality of DNA. Additional pathology material was not available for us to repeat PCR analyses for *IgH* or immunoglobulin light chains. The false-negative *IgH* rearrangement is well known in B-cell lymphomas with translocations involving *IgH*. In such scenarios, *V*, *D* and *J* segments are disrupted. In our case, both *MYC* and *BCL6* had *IgH* as the fusion partners. Another well-documented possibility for the false-negative *IgH* analyses is the presence of extensive somatic hypermutations of *IgH* gene prevents PCR amplification (30). This possibility is supported by the findings of BCL6 negativity by immunohistochemistry in the relapsed tumor despite the presence of *BCL6* rearrangement. However, both initial and relapsed tumors showed *MYC* and *BCL6* rearrangements, supporting that both tumors share a common precursor lesion but diverge to two different histologic subtypes of tumors at initial diagnosis and at relapse, somewhat analogous to acute leukemia switching between myeloid and lymphoid lineages, or analogous to branch evolution of FL (3, 4, 31, 32).

## Conclusion

In summary, we report here a case of HGBL with *MYC* and *BCL6* rearrangements switching to high-grade plasmablastic neoplasm with *MYC* and *BCL6* rearrangements. This is the first report of aggressive mature B-cell neoplasm switching from one histologic category to another, supporting the notion that fully differentiated mature B-cell lymphoma retains plasticity, and expanding the spectrum of lineage or phenotypic switch in B-cell lymphoma. Being aware of this particular scenario will help improve our understanding of the underlying mechanisms of tumor escape from chemotherapy and develop novel anti-tumor therapy.

## Data Availability Statement

The original contributions presented in the study are included in the article/supplementary material. Further inquiries can be directed to the corresponding author.

## Ethics Statement

The studies involving human participants were reviewed and approved by IRB Committee at MD Anderson Cancer Center. Written informed consent for participation was not required for this study in accordance with the national legislation and the institutional requirements.

## Author Contributions

HL and SH wrote the manuscript. QS and JC prepared the data. All authors contributed to the article and approved the submitted version.

## Conflict of Interest

The authors declare that the research was conducted in the absence of any commercial or financial relationships that could be construed as a potential conflict of interest.

## Publisher’s Note

All claims expressed in this article are solely those of the authors and do not necessarily represent those of their affiliated organizations, or those of the publisher, the editors and the reviewers. Any product that may be evaluated in this article, or claim that may be made by its manufacturer, is not guaranteed or endorsed by the publisher.

## References

[1] Quintanal-Villalonga ÁVerifytat Chan JM Yu HA Pe’er D Sawyers CL Sen T. Lineage Plasticity in Cancer: A Shared Pathway of Therapeutic Resistance. Nat Rev Clin Oncol (2020) 17(6):360–71. doi: 10.1038/s41571-020-0340-z PMC7397755 32152485

[2] Thankamony AP Subbalakshmi AR Jolly MK Nair R. Lineage Plasticity in Cancer: The Tale of a Skin-Walker. Cancers (Basel) (2021) 13(14):3602. doi: 10.3390/cancers13143602 34298815 PMC8306016

[3] Liao W Kohler ME Fry T Ernst P. Does Lineage Plasticity Enable Escape From CAR-T Cell Therapy. Lessons from MLL-r leukemia. Exp Hematol (2021) 100:1–11. doi: 10.1016/j.exphem.2021.07.002 34298117 PMC8611617

[4] Schultz L Gardner R. Mechanisms of and Approaches to Overcoming Resistance to Immunotherapy. Hematol Am Soc Hematol Educ Program (2019) 2019(1):226–32. doi: 10.1182/hematology.2019000018 PMC6913466 31808880

[5] Feldman AL Arber DA Pittaluga S Martinez A Burke JS Raffeld M. Clonally Related Follicular Lymphomas and Histiocytic/Dendritic Cell Sarcomas: Evidence for Transdifferentiation of the Follicular Lymphoma Clone. Blood (2008) 111(12):5433–9. doi: 10.1182/blood-2007-11-124792 PMC2424145 18272816

[6] Shao H Xi L Raffeld M Feldman AL Ketterling RP Knudson R. Clonally Related Histiocytic/Dendritic Cell Sarcoma and Chronic Lymphocytic Leukemia/Small Lymphocytic Lymphoma: A Study of Seven Cases. Mod Pathol (2011) 24(11):1421–32. doi: 10.1038/modpathol.2011.102 PMC3175277 21666687

[7] Wang E Papalas J Hutchinson CB Kulbacki E Huang Q Sebastian S. Sequential Development of Histiocytic Sarcoma and Diffuse Large B-Cell Lymphoma in a Patient With a Remote History of Follicular Lymphoma With Genotypic Evidence of a Clonal Relationship: A Divergent (Bilineal) Neoplastic Transformation of an Indolent B-Cell Lymphoma in a Single Individual. Am J Surg Pathol (2011) 35(3):457–63. doi: 10.1097/PAS.0b013e3182098799 21317718

[8] West DS Dogan A Quint PS Tricker-Klar ML Porcher JC Ketterling RP. Clonally Related Follicular Lymphomas and Langerhans Cell Neoplasms: Expanding the Spectrum of Transdifferentiation. Am J Surg Pathol (2013) 37(7):978–86. doi: 10.1097/PAS.0b013e318283099f 23759932

[9] Farris M Hughes RT Lamar Z Soike MH Menke JR Ohgami RS. Histiocytic Sarcoma Associated With Follicular Lymphoma: Evidence for Dramatic Response With Rituximab and Bendamustine Alone and a Review of the Literature. Clin Lymphoma Myeloma Leuk (2019) 19(1):e1–8. doi: 10.1016/j.clml.2018.10.004 30396823

[10] Péricart S Waysse C Siegfried A Struski S Delabesse E Laurent C. Subsequent Development of Histiocytic Sarcoma and Follicular Lymphoma: Cytogenetics and Next-Generation Sequencing Analyses Provide Evidence for Transdifferentiation of Early Common Lymphoid Precursor-a Case Report and Review of Literature. Virchows Arch (2020) 476(4):609–14. doi: 10.1007/s00428-019-02691-w 31807922

[11] Fraser CR Wang W Gomez M Zhang T Mathew S Furman RR. Transformation of Chronic Lymphocytic Leukemia/Small Lymphocytic Lymphoma to Interdigitating Dendritic Cell Sarcoma: Evidence for Transdifferentiation of the Lymphoma Clone. Am J Clin Pathol (2009) 132(6):928–39. doi: 10.1309/AJCPWQ0I0DGXBMHO 19926586

[12] Burger JA Landau DA Taylor-Weiner A Bozic I Zhang H Sarosiek K. Clonal Evolution in Patients With Chronic Lymphocytic Leukaemia Developing Resistance to BTK Inhibition. Nat Commun (2016) 20 7:11589. doi: 10.1038/ncomms11589 PMC4876453 27199251

[13] Rassidakis GZ Stromberg O Xagoraris I Jatta K Sonnevi K. Trametinib and Dabrafenib in Histiocytic Sarcoma Transdifferentiated From Chronic Lymphocytic Leukemia With a K-RAS and a Unique BRAF Mutation. Ann Hematol (2020) 99(3):649–51. doi: 10.1007/s00277-020-03941-7 32009180

[14] Hure MC Elco CP Ward D Hutchinson L Meng X Dorfman DM. Histiocytic Sarcoma Arising From Clonally Related Mantle Cell Lymphoma. J Clin Oncol (2012) 30(5):e49–53. doi: 10.1200/JCO.2011.38.8553 22184374

[15] Wang E Hutchinson CB Huang Q Sebastian S Rehder C Kanaly A. Histiocytic Sarcoma Arising in Indolent Small B-Cell Lymphoma: Report of Two Cases With Molecular/Genetic Evidence Suggestive of a 'Transdifferentiation' During the Clonal Evolution. Leuk Lymphoma (2010) 51(5):802–12. doi: 10.3109/10428191003699845 20331331

[16] Ambrosio MR De Falco G Rocca BJ Barone A Amato T Bellan C. Langerhans Cell Sarcoma Following Marginal Zone Lymphoma: Expanding the Knowledge on Mature B Cell Plasticity. Virchows Arch (2015) 467(4):471–80. doi: 10.1007/s00428-015-1814-8 26286813

[17] Sabatini PJB Tremblay-LeMay R Ahmadi Moghaddam P Delabie JMA Sakhdari A. Marginal Zone Lymphoma Transdifferentiated to Histiocytic Sarcoma. Br J Haematol (2021) 194(6):1090–4. doi: 10.1111/bjh.17582 34096049

[18] Zhang Q Orlando EJ Wang HY Bogusz AM Liu X Lacey SF. Transdifferentiation of Lymphoma Into Sarcoma Associated With Profound Reprogramming of the Epigenome. Blood (2020) 136(17):1980–3. doi: 10.1182/blood.2020005123 PMC7582558 32518951

[19] Kawashima I Oishi N Kasai K Inoue T Hosokawa E Nakadate A. Transdifferentiation of Mantle Cell Lymphoma Into Sarcoma With Limited Neuromuscular Differentiation After Conventional Chemotherapy. Virchows Arch (2021) 9. doi: 10.1007/s00428-021-03148-9 34226971

[20] Trecourt A Mauduit C Szablewski V Fontaine J Balme B Donzel M. Plasticity of Mature B Cells Between Follicular and Classic Hodgkin Lymphomas: A Series of 22 Cases Expanding the Spectrum of Transdifferentiation. Am J Surg Pathol (2021). doi: 10.1097/PAS.0000000000001780 34265801

[21] Aussedat G Traverse-Glehen A Stamatoullas A Molina T Safar V Laurent C. Composite and Sequential Lymphoma Between Classical Hodgkin Lymphoma and Primary Mediastinal Lymphoma/Diffuse Large B-Cell Lymphoma, a Clinico-Pathological Series of 25 Cases. Br J Haematol (2020) 189(2):244–56. doi: 10.1111/bjh.16331 32030731

[22] Kridel R Mottok A Farinha P Ben-Neriah S Ennishi D Zheng Y. Cell of Origin of Transformed Follicular Lymphoma. Blood (2015) 126(18):2118–27. doi: 10.1182/blood-2015-06-649905 PMC4626253 26307535

[23] Yuan S Norgard RJ Stanger BZ. Cellular Plasticity in Cancer. Cancer Discovery (2019) 9(7):837–51. doi: 10.1158/2159-8290.CD-19-0015 PMC6606363 30992279

[24] Lucas DR Shukla A Thomas DG Patel RM Kubat AJ McHugh JB. Dedifferentiated Liposarcoma With Inflammatory Myofibroblastic Tumor-Like Features. Am J Surg Pathol (2010) 34(6):844–51. doi: 10.1097/PAS.0b013e3181db34d8 20431481

[25] Ferreira I Droop A Edwards O Wong K Harle V Habeeb O. The Clinicopathologic Spectrum and Genomic Landscape of De-/Trans-Differentiated Melanoma. Mod Pathol (2021) 34(11):2009–19. doi: 10.1038/s41379-021-00857-z 34155350

[26] Geyer JT Ferry JA Longtine JA Flotte TJ Harris NL Zukerberg LR. Characteristics of Cutaneous Marginal Zone Lymphomas With Marked Plasmacytic Differentiation and a T Cell-Rich Background. Am J Clin Pathol (2010) 133(1):59–69. doi: 10.1309/AJCPW64FFBTTPKFN 20023259

[27] Swerdlow SH Kuzu I Dogan A Dirnhofer S Chan JKC Sander B. The Many Faces of Small B Cell Lymphomas With Plasmacytic Differentiation and the Contribution of MYD88 Testing. Virchows Arch (2016) 468(3):259–75. doi: 10.1007/s00428-015-1858-9 PMC5002945 26454445

[28] Klein U Dalla-Favera R. Germinal Centres: Role in B-Cell Physiology and Malignancy. Nat Rev Immunol (2008) 8(1):22–33. doi: 10.1038/nri2217 18097447

[29] Basso K Dalla-Favera R. Germinal Centres and B Cell Lymphomagenesis. Nat Rev Immunol (2015) 15(3):172–84. doi: 10.1038/nri3814 25712152

[30] Langerak AW Groenen PJ Bruggemann M Beldjord K Bellan C Bonello L. EuroClonality/BIOMED-2 Guidelines for Interpretation and Reporting of Ig/TCR Clonality Testing in Suspected Lymphoproliferations. Leukemia (2012) 26(10):2159–71. doi: 10.1038/leu.2012.246 PMC3469789 22918122

[31] Pasqualucci L Khiabanian H Fangazio M Vasishtha M Messina M Holmes AB. Genetics of Follicular Lymphoma Transformation. Cell Rep (2014) 6(1):130–40. doi: 10.1016/j.celrep.2013.12.027 PMC4100800 24388756

[32] Okosun J Montoto S Fitzgibbon J. The Routes for Transformation of Follicular Lymphoma. Curr Opin Hematol (2016) 23(4):385–91. doi: 10.1097/MOH.0000000000000255 27135979

